# Supplementary material for: Succinate induces skeletal muscle fiber remodeling via SUNCR1 signaling
Source: EMBO Rep. 2019 Jul 18;20(9):e47892. doi: 10.15252/embr.201947892 (PMC6727026; doi:10.15252/embr.201947892)
Supplement: Supplementary file 1 — Expanded View Figures PDF [file EMBR-20-e47892-s001.pdf]

Expanded View Figures

Figure EV1. Effects of succinate on growth performance and muscle fiber composition in mice (related to Figs 1 and 3).

Male C57BL/6J mice were fed with chow diet supplemented with 0, 0.5%, or 1% SUC for 8 weeks.

A, B (A) Cumulative food intake and (B) liver index of mice treated with SUC for 8 weeks (n = 8).

C, D Immunoblots and quantification of p-mTOR, mTOR, p-FoxO3a, FoxO3a, p-AKT, and AKT protein in gastrocnemius (n = 3).

E–H Representative images and quantification of laminin (green), MyHC I (red), and MyHC IIb (red) immunofluorescent staining in the (E, F) soleus and (G, H) extensor digitorum longus muscle. The graphs show the MyHC I and MyHC IIb fiber ratios (n = 6). Scale bar in (E, G) represents 100 μm.

I–N The percentage of SDH positive in the (I, J) gastrocnemius, (K, L) soleus, and (M, N) extensor digitorum longus muscle is shown by SDH enzyme staining. Only darkly stained SDH fibers are treated as SDH-positive fibers. The graphs show the SDH-positive fiber ratios (n = 4–6). Scale bar in I, K, and M represents 100 μm.

Data information: Results are presented as mean ± SEM. Different letters between bars mean $P \leq 0.05$ in one-way ANOVA analyses followed by *post hoc* Tukey's tests.

© 2019 The Authors

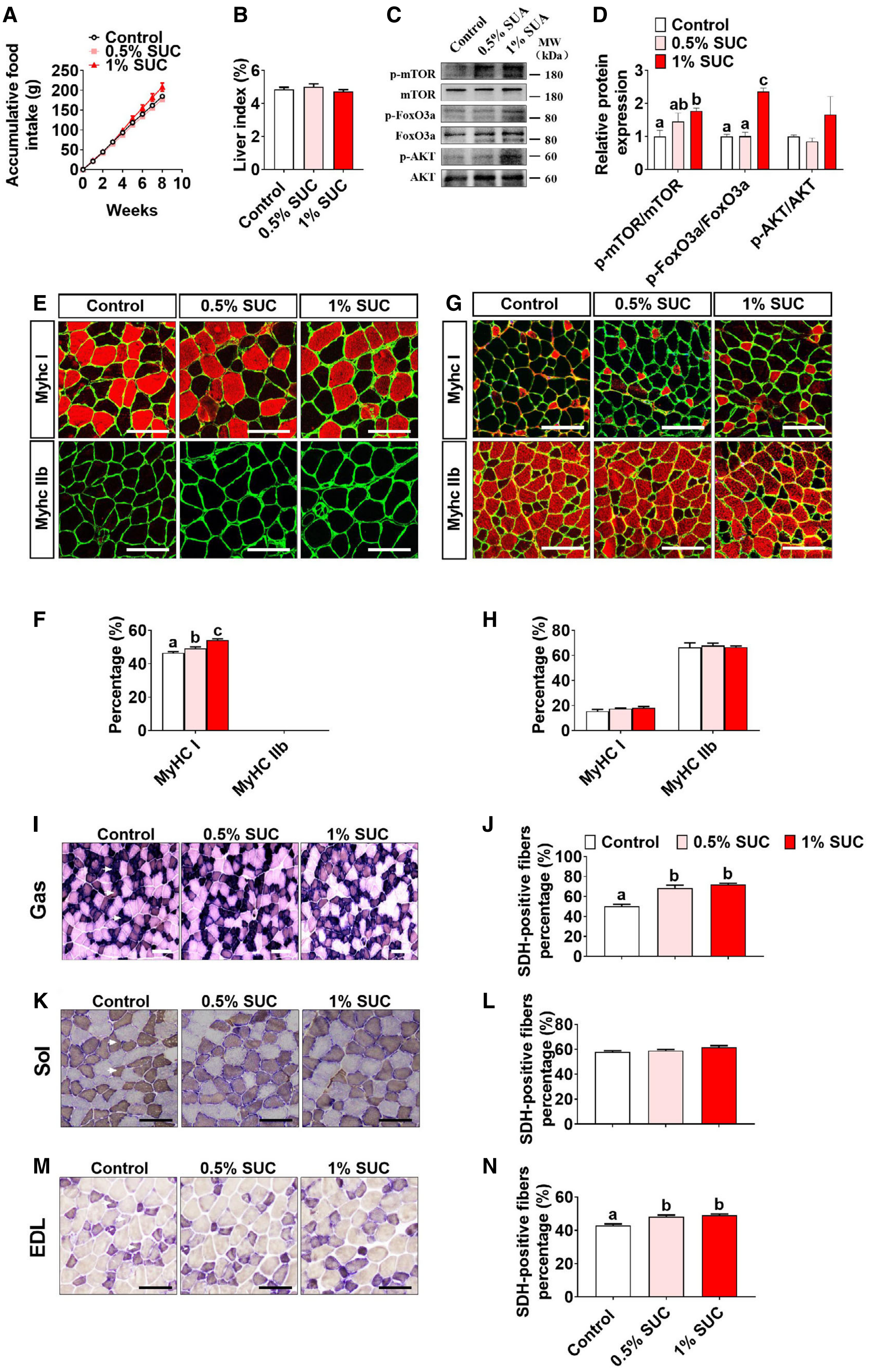

Figure EV1.

 © 2019 The Authors

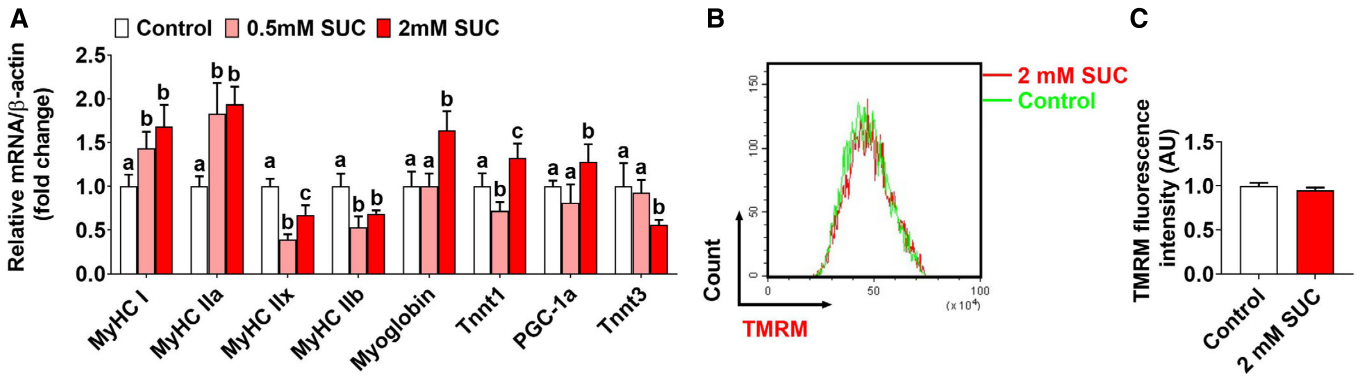

Figure EV2. Effects of succinate on muscle fiber and mitochondrial function of C2C12 cells (related to Fig 5).

C2C12 cells were treated with 0, 0.5 mM, or 2 mM SUC for 48 h.

A The mRNA expression of MyHC I, MyHC IIa, PGC-1α, myoglobin, TnnT1 MyHC IIb, MyHC IIx, and TnnT3 in C2C12 cells.

B, C Fluorescence-activated cell sorting (FACS) analysis of TMRM fluorescence intensity and the relative mean fluorescence intensity of TMRM.

Data information: Results are presented as mean ± SEM (n = 5–6). Different letters between bars mean $P \leq 0.05$ in one-way ANOVA analyses followed by *post hoc* Tukey's tests.

© 2019 The Authors

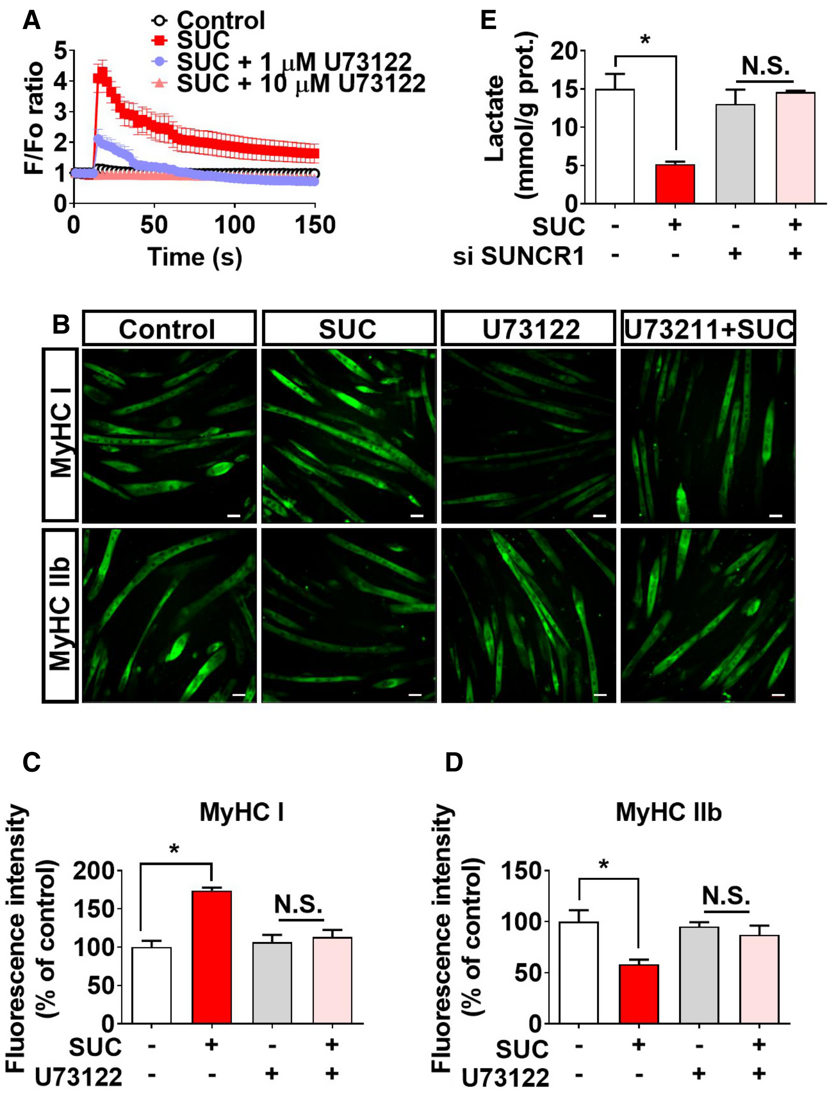

Figure EV3. Role of SUNCR1/PLC-β in succinate-induced *in vitro* fiber-type transition in myotubes (related to Fig 6).

A [Ca^{2+}]i of C2C12 cells treated with vehicle, SUC (2 mM), SUC (2 mM) + PLC-β inhibitor U73122 (1 μM), or SUC (2 mM) + PLC-β inhibitor U73122 (10 μM; *n* = 9–10).

B–D After 6 days of differentiation, C2C12 cells were treated with vehicle, SUC (2 mM), U73122 (5 μM), or SUC (2 mM) + U73122 (5 μM) for 48 hrs. Representative images (C) and (D, E) quantification of MyHC I and MyHC IIb immunofluorescent staining (green) in the C2C12 cells (*n* = 3). Scale bar in (C) represents 50 μm.

E C2C12 cells were transfected with vector or siSUNCR1, cultured for 6 days in a differentiation medium, and then treated with SUC (2 mM) for 48 h to test the concentration of lactic acid in medium (*n* = 5–6).

Data information: Results are presented as mean ± SEM. *$P \leq 0.05$ by non-paired Student's *t*-test.

 © 2019 The Authors

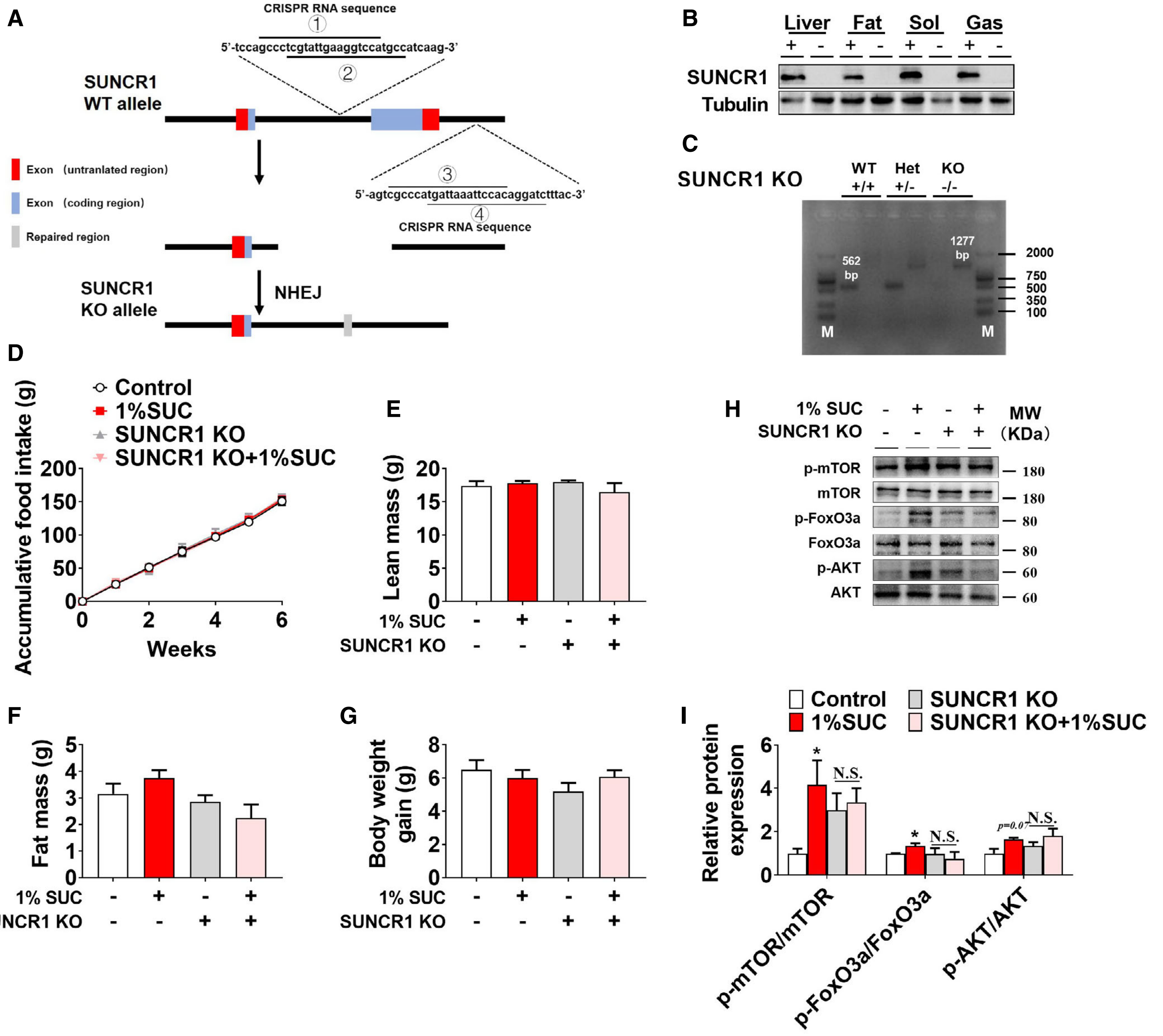

Figure EV4. SUNCR1 global knockout blocks the effect of succinate on protein synthesis (related to Fig 7).

A Schematic representation of SUNCR1 KO by Clustered Regularly Interspaced Short Palindromic Repeats (CRISPR) strategy. The sgRNA sites were located in intron 1 and intron 2 of SUNCR1 gene. Four sgRNAs were designed to delete exon 2 of SUNCR1 gene. The DNA sequences contained sgRNA-binding regions are labeled with lines.

B Immunoblots of SUNCR1 protein in liver, fat, soleus (sol), and gastrocnemius (gas) from WT and SUNCR1 KO mice.

C Representative images for genotyping screen of WT, heterozygous (Het), and homozygous (KO) SUNCR1 KO mice.

D–G (D) Cumulative food intake, (E) lean mass, (F) fat mass, and (G) body weight gain of WT or SUNCR1 KO mice after 6 weeks of dietary supplementation of 0 or 1% SUC.

H, I Immunoblots and quantification of p-mTOR, mTOR, p-FoxO3a, FoxO3a, p-AKT, and AKT proteins in gastrocnemius from WT or SUNCR1 KO mice after 6 weeks of dietary supplementation of 0 or 1% SUC ($n = 3$).

Data information: Results are presented as mean ± SEM ($n = 5$–6). *$P \le 0.05$ by non-paired Student's t-test.

© 2019 The Authors

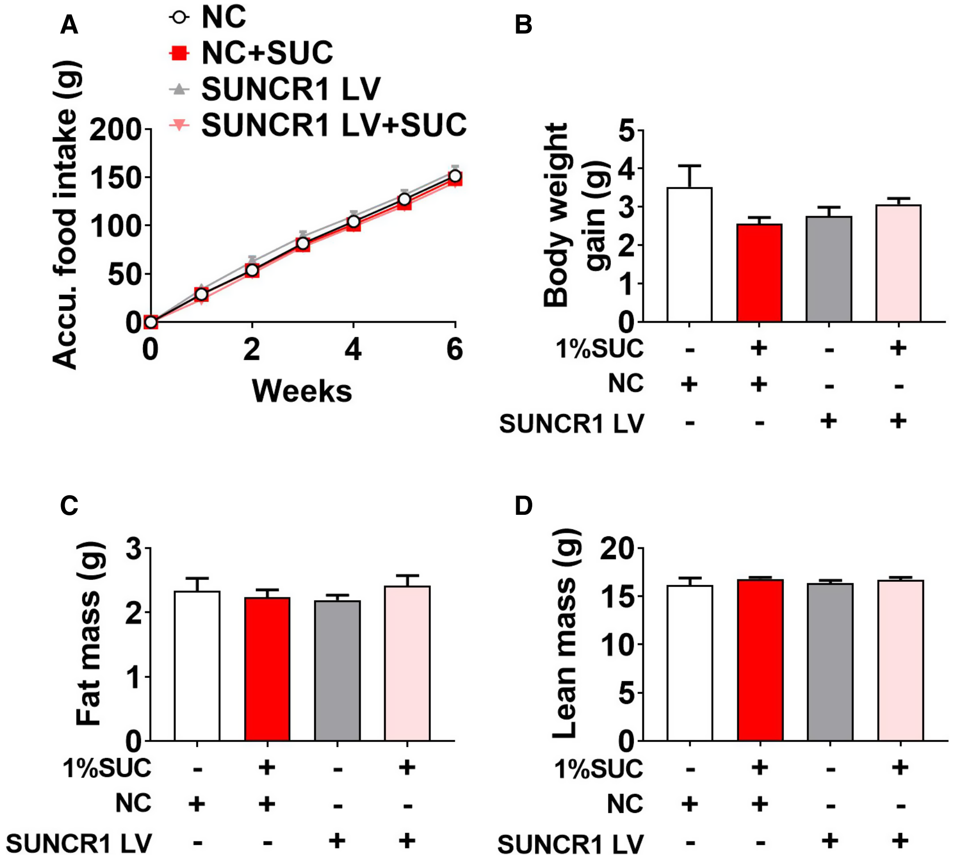

Figure EV5. Effects of gastrocnemius-specific SUNCR1 knockdown on body weight (related to Fig 8).

A–D Male C57BL/6J mice were injected with LV-shScrambled or shSUNCR1 lentivirus specifically into the gastrocnemius at 6 weeks of age. After 2 weeks of recovery, mice were fed with chow diet supplemented with 0 or 1% SUC for 6 weeks. (A) Cumulative food intake, (B) body weight gain, (C) fat mass, and (D) lean mass of mice after 6 weeks of dietary SUC supplementation.

Data information: Results are presented as mean ± SEM (n = 7–8).

 © 2019 The Authors
