# Supplementary material for: Succinate induces skeletal muscle fiber remodeling via SUNCR1 signaling
Source: EMBO Rep. 2019 Jul 18;20(9):e47892. doi: 10.15252/embr.201947892 (PMC6727026; doi:10.15252/embr.201947892)
Supplement: Supplementary file 2 — Table EV1 [file EMBR-20-e47892-s002.docx]

Succinate Induces Skeletal Muscle Fiber Remodeling via SUNCR1 Signaling Pathway

**Tao Wang^1*^, Ya-Qiong Xu^1*^, Ye-Xian Yuan^1*^, Ping-Wen Xu^3^,Cha Zhang^1^, Fan Li^1,^ Li-Na Wang^1^, Cong Yin^1^, Lin Zhang^1^, Xing-Cai Cai^1^, Can-Jun Zhu^1^, Jing-Ren Xu^1^, Bing-Qing Liang^1^, Sarah Schaul^3^, Pei-Pei Xie^1^, Dong Yue^1^, Zheng-Rui Liao^1^, Lu-Lu Yu^1^, Lv Luo^1^, Gan Zhou^1^, Jin-Ping Yang^1^, Zhi-hui He^1^, Man Du^1^, Yu-Ping Zhou^1^, Bai-Chuan Deng^1^, Song-Bo Wang^1^, Ping Gao^1^, Xiao-Tong Zhu^1^, Qian-Yun Xi^1^, Yong-Liang Zhang ^1^, Gang Shu^1,2#^ and Qing-Yan Jiang^1,2^**

**Expanded View Table**

Table EV1. PCR primer sequences of related genes

| Gene abbreviation | Forward Primer （5’-3’） | Reverse Primer （5’-3’） |
| --- | --- | --- |
| Sucnr1 | TGGTTGGCAACAGAGGCTAT | TGACATTCCCAAGCAGTCCA |
| MyHC I | CCTTGGCACCAATGTCCCGGCTC | CCTTGGCACCAATGTCCCGGCTC |
| MyHC IIa | ATGAGCTCCGACGCCGAG | TCTGTTAGCATGAACTGGTAGGCG |
| MyHC IIb | TGATCACCACCAACCCAT | CAGCCTTGTCAGCAACTTC |
| MyYC IIx | AAGGAGCAGGACACCAGCGCCCA | ATCTCTTTGGTCACTTTCCTGCT |
| PGC-1α | TATGGAGTGACATAGAGTGTGCT | CCACTTCAATCCACCCAGAAAG |
| Myoglobin | ACACGCCACCAAGCACAAG | CCTGGAAGCCTAGCTCCTTGTAC |
| Tnnt1 | ACTGCCTCCCGTAAACTCATG | TCCTCATCCACCACCTCTACC |
| Tnnt3 | CCCTCATTGACAGCCACTT | CAATCTGTTCTGGCGTTCC |
| ATPase6 | CAAACAAATAATGCTAATCCA | GCTGTAAGCCGGACTGCTAAT |
| mit1000 | CGATAAACCCCGCTCTACCT | AGCCCATTTCTTCCCATTTC |
| Cyt b | AACATACGAAAAACACACCCATT | AGTGGCGACCTGTAAGAAAATGTGTT |
| COX2 | AGTTGATAACCGAGTCGTTCTG | CTGTTGCTTGATTTAGTCGGC |
| Uqcrfs1 | TACAGATGTCAAGGTGCCCG | TTTGGCCGCATAAGCAACAC |
| Ndufa1 | TTATGGGGGTGTGCTTGGTC | GTTTTTCCTTGCCCCCGTTG |
| Cox5a | TGTCTGTTCCATTCGCTGCT | AACCGTCTACATGCTCGCAA |
| β-globin | GAAGCGATTCTAGGGAGCAG | GGAGCAGCGATTCTGAGTAGA |
